# Humanitarian observatories: insights for reforming humanitarianism from below

**DOI:** 10.1186/s41018-025-00172-1

**Published:** 2025-06-03

**Authors:** Dorothea Hilhorst, Kaira Zoe Alburo-Cañete, Juan Ricardo Aparicio, Patrick Milabyo Kyamusugulwa, Tadesse Kassa Woldetsadik

**Affiliations:** 1 International Institute of Social Studies, Erasmus University, Rotterdam, the Netherlands; 2 Universidad de los Andes, Bogotá, Colombia; 3 Institut Superieur de Developpement Rural, Bukavu, Democratic Republic of the Congo; 4 Addis Ababa University, Addis Ababa, Ethiopia

## Abstract

In the last few decades, there has been a marked turn to “humanitarianism from below” in thinking about and organizing humanitarian action, which is among other expressed in the localization agenda of humanitarian action. In the last years, there have been many initiatives to strengthen national actors as well as initiatives that are directed to organization, advocacy, and collective action. This paper theoretically positions the role of national and local service providers in the humanitarian arena and politics of knowledge production and then presents a specific initiative of humanitarian observatories in three countries. The paper brings out a number of issues relevant for other initiatives aiming to strengthen the role of national and local actors, namely that humanitarians are not the only relevant actors to deal with humanitarian crises, that context matters, the importance of agenda-setting, and the importance of sideways interaction between observatories in different crisis-affected regions.

## Introduction

In the last few decades, there has been a marked turn to “humanitarianism from below” in thinking about and organizing humanitarian action. Thirty years ago, the Code of Conduct for the International Red Cross and Red Crescent Movement and Nongovernmental Organizations (NGOs) in Disaster Relief was launched. Two articles of the code particularly stood out: “we shall attempt to build disaster response on local capacities” and “ways shall be found to involve program beneficiaries in the management of relief aid’.^1^ If nothing else, this code revealed the western-centric nature of aid: the “we” referred to in the article was limited to international humanitarian agencies. It also showed the wavering attitude that these actors had in relation to the largely unexplored practices of working with, let alone following the lead of the “local capacities” and the “beneficiaries” for whom their good was being done. This language was especially remarkable considering that many of the INGO signatories were development agencies with a long record of participation and partnership with “local capacities,” who were appreciated for their capacities as well as their knowledge and expertise. The Red Cross Movement moreover largely consisted of national societies. This was apparently no obstacle to signing the code, and publications of those years rarely challenged the inequalities and power differentials embedded in its language.

In 2016, the first ever World Humanitarian Summit not only reflected changing values in relation to national actors of the humanitarian world but also it acted as a vehicle to promote this shift. It introduced the agenda of “localization of aid,” which aims to better respect national authorities and agencies and enhance their role in the implementation and decision-making of aid as part of the “participation revolution” the Summit sought to engender. One of the most tangible outcomes of the summit was the pledge of donors (united in the Grand Bargain) to commit to more direct funding of national actors (UNGA 2016).

The introduction of the localization agenda was not without critique (Mulder 2023). It was not equally embraced by all humanitarians, some of whom would retain their fear that national actors would not live by the cherished principles of neutrality and impartiality. Others would maintain over the years that the localization agenda paid lip-service to aspirations of equality yet was never successfully translated in practice. One of the most eye-catching aspects of critique is that the use of the term “localization” revealed a similar western-centrism as seen in the older generation Code of Conduct. The force that carries out the localizing is international, and hence, this remains the default or standard model of humanitarian action. Localization is presented as a gift bestowed on national actors, a gracious act of power-sharing, rather than a realization that power must be restored to where it belongs, with the people that experience the crisis that needs relieving (see also Roepstorff 2020). This criticism can be recognized among voices calling for the decolonization of aid (Aloudat & Khan 2022; Foreign Aid Is Having a Reckoning 2021). Notwithstanding the aversion many critical scholars have for the term localization, it seems to be stuck and continues to be used widely. We are set to avoid the term and speak instead in the remainder of this paper about advancing the roles of national actors.

The case for advancing the role of national actors has different grounds. Firstly, it is based on ethical and indeed legal considerations. Sovereign national governments and authorities are in the lead of crisis response, and the international community should only have an auxiliary and supporting role to play. This is not how the relation has evolved in practice, however, and especially since the early 1990 s — when international humanitarian machinery vastly expanded — humanitarians have tended to assume a much more central role (Harvey, 2009). Since the turn of the twenty-first century, governments have become more assertive in claiming back their space, starting with the Gujarat earthquake in India. Humanitarian actors have a variety of strategies to collaborate with governments, depending on the level of involvement of the government in the crisis when it, for example, stems from conflict and the political will and capacities that governments display in ensuring that aid reaches people most in need.

The ethical case centers on reducing inequalities in the sector, particularly in working relationships with national or local service providers. National and local actors play major roles in humanitarian service delivery as employees of international agencies (at a lesser paygrade than their expatriate colleagues) or as part of a national agency or nongovernmental organization. In a study conducted on wage disparities between international and national aid workers, for instance, researchers showed that local staff were paid four times less on average compared to their expatriate counterparts. Such disparities are not limited to salaries but include as well allowances for accommodations, transportation, and other benefits (Carr & McWha-Hermann 2016). In addition, concerns over “unethical recruitment practices” by international organizations have surfaced in recent years. Staff poaching has often resulted in smaller organizations and local governments losing human resources and homegrown talent to international agencies, which adversely affects their sustainability (McCommon & Sutton 2023). Ironically, it is these same organizations who work with, and support, affected communities before international agencies arrive and long after they exit. Hence, there is a growing movement among various international humanitarian networks to put in place frameworks and guidelines to shift these practices towards valuing national actors in terms of both wage parity and ethical recruitment (ibid). Moreover, issues around restoring national actors’ autonomy in relation to international donor agendas (e.g., calls for “shifting the power”) have also surfaced as important ethical issues in humanitarian aid.^2^

Secondly, the case for recognizing and advancing the role of national actors is built on functional and pragmatic grounds. Humanitarian needs are growing. Currently, 38 countries are engaged in conflict, and at the same time, we see a steep incline in the number of disasters adversely affecting vulnerable people’s livelihoods. Many expect that the impact of climate change will lead to an intensification and multiplication of humanitarian crisis situations in the near future (IFRC 2020). More than 300 million people (1 in 27) currently depend on humanitarian assistance (UNOCHA 2023). Although humanitarian budgets continue to grow, needs risk outgrowing capacities of global humanitarian response. In seeking to stretch its boundaries to accommodate crisis response wherever it is needed, one proposed solution would be to rely more on national service delivery capacities.

In recent years, there have been many initiatives to strengthen national actors as well as initiatives that are directed to organization, advocacy, and collective action. The turn towards national service delivery as a key resource in responding to humanitarian crises comes about from above as much as from below, where national actors are increasingly claiming space. These initiatives have usually been founded to unite and amplify southern voices to increase their influence in the global humanitarian system. Notable initiatives include the Charter4 Change, which was launched at the World Humanitarian Summit in 2016 to promote the role of national actors. It comprises a mix of international and national organizations. Another successful initiative is the Southern NGO network NEAR, which was also formed in the prelude of the summit. The START Network is also a key player in advancing locally led humanitarian action and rapid financing, e.g., through the establishment of area-based pooled funds. These initiatives have also focused on making southern agencies and expertise more visible to enhance their position in obtaining contracts. The International Humanitarian Studies Association, for example, together with The NEAR network and Tufts University maintains a database of organizations and individuals and their expertise and countries to facilitate recruitment for consultancy work or other humanitarian assignments.^3^

These recent developments within the sector highlight the need to seek insights into the roles of national actors in humanitarian action, how their experiences reveal different knowledges and practices that are often overlooked (and sometimes dismissed), and the potentials for such actors to advocate for reforms in their own context and perhaps even beyond. Along these lines, this paper discusses a recent movement that has the explicit objective to learn, share, and advocate to reform humanitarianism at the *national* level. It concerns a network of humanitarian observatories, which can broadly be defined as groups comprising a variety of national or regional actors that discuss and reflect on trends in humanitarian action in situ and propose/advance reforms when required. The authors of this paper have established these networks in the Democratic Republic of Congo, Ethiopia, Latin America and the Caribbean, and the Philippines. This has been done as part of a research consortium on humanitarian governance (referred to as the Hum-Gov project).^4^ The network has now spread to several more countries, currently including South Asia (based in India), Namibia, Libya, Central and Eastern Europe (based in Poland), Somalia, the Netherlands, and Pakistan.

The paper will first position the role of national service providers theoretically. We step away from both the normative and pragmatic notions of why the role of national actors must be strengthened and take a more empirical approach to how humanitarian action contextually evolves as the interplay of a multitude of actors. To do this, the article anchors its claims around the concepts of the *humanitarian arena* and *politics of knowledge* which underpin the inspiration, development, and design of the humanitarian observatories. We will then tell the story of the observatories as they have evolved in their own context, with a focus on emerging insights that can strengthen the case for reforming humanitarianism from below. We do so by presenting a conversation between us (the authors) — illuminating the different approaches to establishing observatories, embodying the aspirations embedded in these endeavors, and articulating hopes as well as concerns for the future of the observatories individually and as a network. Using these actual words in a conversation as a method of presenting our ideas provides insights into the lived experiences of the observatories and also reveals the spirit of exchange and learning that have shaped our approaches to network building.

## The humanitarian arena and politics of knowledge

In 2010, Dorothea Hilhorst and Bram Jansen laid out the foundation of an arena perspective of humanitarian action. The basis of this perspective is the notion that humanitarian action, including processes of implementation and policy-making, is shaped in practice. Humanitarian action is considered as an “arena where a multitude of actors, including humanitarians and the disaster-affected recipients of aid, shape the everyday realities of humanitarian action” (Hilhorst & Jansen 2013, p. 1117). The realities and outcomes of aid depend on how actors along and around the aid chain — donor representatives, headquarters, field staff, affected communities, and surrounding actors — use their social agency to interpret the context, the needs, their own role, and each other. At the time, the meaning of “humanitarian” was mainly restricted to international humanitarian agencies. The arena perspective intended to broaden this understanding by asking empirically how the conditions of service delivery in crisis situations are shaped in practice and by recognizing that this implies many different actors.

In the last decade, the recognition of plurality in humanitarian service delivery has become paramount. This is reflected in the growing literature on the roles of NGOs that do not necessarily claim the label of “humanitarian” (in the traditional sense) as well as the literature on citizen-based or vernacular humanitarianisms (Ager et al. 2015; Brković, 2023; Fechter & Schwittay 2019; Richey 2018; Rozakou 2017). In particular, the increasing visibility of the role of mutual aid networks as a citizen-driven response to crises, built on culturally specific forms of reciprocity, is worth noting. We find various manifestations of these solidarity-based support networks in different contexts: the establishment of emergency response rooms in Sudan (Nasir et al. 2023), organizing of *Centros de Apuyo Motuo* (mutual support centers) in Puerto Rico after Hurricane Maria (Velez-Velez & Villarrubia-Mendoza 2021) and the proliferation of community pantries across the Philippines during the COVID pandemic (Abesamis et al. 2022; Del Castillo 2021), and many more (Carstensen et al. 2021; El Zerbi et al. 2022; Viga & Refstie 2024).

In the humanitarian arena, attention is also paid to the strategizing and constructive roles of affected people in shaping humanitarian aid. People in need strategize to reach humanitarian agencies and become eligible for their services while aiming to mold those services to their needs. This encompasses more than the accountability, participation, and feedback mechanisms that aid agencies build into their programs. People claim spaces in visible and invisible ways. A classic example is the aid recipient that sells received goods on the market because these do not fit their needs or people opting out of programs they find irrelevant and hence “vote with their feet” (Gaventa et al. 2023). People are not solely defined by their needs and are part of stratified and diverse communities, having different identities, playing different roles, and taking part in more or less powerful networks. Without denying the dependency people may have on outside assistance for their survival, it is important to consider how communities deal with crises and aim to obtain assistance they need and how they co-shape humanitarian action.

The arena perspective is furthermore sensitive to the different actors and factors that drive the definitions and conditions of crises as well as the responses to crises. Humanitarian practices, therefore, can be understood as activities of the international humanitarian machinery as well as the multiple ways in which groups and institutions (e.g., formal, informal, local/national, international, government, non-government) interact, address, and respond to situations of crisis. This broadening of scope opens possibilities for recognizing this plurality of form and approaches as well as its embeddedness in context.

The complexity of humanitarian action extends beyond modes of coordination, actors involved, and the forms of participation enabled. It is also deeply embedded in the practices of knowledge production. The collection of information about crisis-affected populations has become increasingly central to decision-making processes related to aid provision and organizational strategies. This data not only informs interventions but also influences how crisis-affected individuals and their environments are represented, imagined, and acted upon. As Potts et al. (2022) observe, knowledge production in these contexts is frequently shaped by power imbalances, entangled in colonial legacies, and perpetuated through extractivist and externally driven methodologies in humanitarian research and data collection (Hilhorst 2020, Alburo-Cañete and Villacis, 2025).

The vision paper *Toward an Equitable Humanitarian Knowledge and Evidence Landscape* (HAG et al. 2024) highlights the limited representation of actors and institutions from the Global South and the undervaluation of local and indigenous knowledge systems as key “sites of inequities” in humanitarian knowledge production. Conventional aid processes are typically driven by international humanitarian organizations and agencies, which collect information about aid recipients while simultaneously shaping humanitarian agendas. Researchers, often from well-resourced institutions in the Global North, dominate the production of knowledge about crisis conditions and the suffering of distant, racialized, and “needy” others, the vast majority of whom are located in the Global South.

A growing body of scholarship (e.g., Gaillard 2019; Van der Haar et al. 2013) has problematized the pervasiveness of extractive, technocentric, and inequitable research practices in crisis settings. These scholars also critique methods that claim to be participatory but often remain tokenistic and lack reflexivity (see also Lenette 2022). For instance, the *Statement of Commitments from Humanitarian Scholars* presented at the 2016 World Humanitarian Summit emphasized the need for more collaborative and inclusive humanitarian research. It recognized “nontraditional knowledge actors and affected communities” as valuable sources and producers of knowledge (IHSA 2016). While these commitments represent positive steps, their implementation has been slow and fraught with challenges (van Duijn & Hilhorst 2019). In light of these issues, there is a pressing need to develop and institutionalize research partnership models that truly embody principles of equity and inclusion. In this way, a more grounded and holistic perspective on humanitarian practices and their governance can also be developed.

The preceding discussion on the humanitarian arena and knowledge production provides conceptual and analytical frames for understanding the complexities of humanitarian action. These perspectives underscore the importance of attending to the plurality of actors, the interplay of power dynamics, and the embeddedness of humanitarian practices within broader sociopolitical and economic contexts. Together, they illuminate how humanitarian action is co-constructed by diverse stakeholders, shaped by systemic inequities, and influenced by the ways crises and affected communities are represented and understood. These frames have been instrumental in grounding our empirical research, guiding our analysis of humanitarian processes, and informing our exploration of more equitable and contextually responsive practices as well as humanitarian research partnerships. The insights these have brought about have been translated into the praxis of the humanitarian observatories.

## Praxis in the design of the observatories

The Hum-Gov project through which the first observatories have been formed aimed to go beyond understanding the endless variations of humanitarian action and contribute to a praxis that is congruent to the theories outlined above. How could their insight be translated and applied in practice? Three main insights that emanated from our own work and numerous relevant and inspiring publications of recent years were the starting point of this application exercise.

1. As humanitarian action varies in different contexts, it is not useful to seek a standard format for humanitarian dialogue and conversations.

Humanitarian action, while often understood as externally driven, takes place within institutional, political, and cultural landscapes that are constituted by specific histories. Navigating these complex landscapes often requires understanding the different relationships between multiple actors, the politics that underpin their interactions, and the cultural groundings that influence how such dialogues and conversations can occur. Hence, finding common ground for conversation is highly contextual and negotiated. Indeed, international agencies have increasingly articulated the need for grounded frameworks and approaches. More recently, this is affirmed through the UNOCHA’s Flagship Initiative, a 3-year project which aims to develop context-specific solutions to humanitarian challenges in a few pilot countries (UNOCHA 2024). However, apart from having these context-focused discussions facilitated by international agencies, having national actors that possess embodied knowledge *leading* these conversations in ways that are appropriate to their settings can provide deeper insights into how humanitarian action takes shape, is contested, and can be reworked.

2. As humanitarian action is shaped in practice, it should also be reformed in practice.

Organizational reform is usually conceived as starting with policy — from the top of the organization and trickling down to its operations. However, reality is often reversed (Colebatch, 2009). It could well be argued that policy is the outcome of changes that have been tried in practice and pushed for by experienced aid workers. As illustrated by the history of localization in the introduction of this paper, the formulation of the policy culminates from experiences, knowledges, and voices from practice. This culmination then imprints itself on practice. If indeed humanitarian action is the embedded outcome of the interplay of actors, it may be more useful to seek to reform governance in its context rather than seeking to develop policy to reform humanitarianism at the global level. This is not to deny the relevance of system change from “above.” It is a plea to give more recognition and space to the potential of change from “below.” Such changes from “below,” even those that are small-scale, spontaneous, or incremental, can also have transformative effects.

3. As humanitarian action is shaped by an amalgam of actors, talking about practice and change should likewise involve a multitude of actors.

Humanitarian crisis is usually seen at the domain of humanitarian actors and authorities. However, since crises emanate from a range of factors and the response to crises affects state-society relations, it follows that a much larger range of actors has a stake in the understanding of and response to crisis. Knowledge, observations, and opinions of citizens matter, whether or not they are directly affected by the crisis. Citizens may not all be part of the formal humanitarian response, but they are often part of the less visible (or made invisible) forms of community responses to crises (Richey 2018; Schwiertz & Schwenken 2020). In many contexts, faith-based groups constitute a significant part of such responses (Ager et al. 2015). Civil society, including in the form of media organizations and academic institutions, also has an important role in the circulation of information that shapes discourses around humanitarian action as well as upholding or challenging the legitimacy of institutions and agencies that respond to crisis. Young people, for example, often utilize social media and other technologies to mobilize resources for crises responses, challenge the status quo, and hold authorities and humanitarians accountable for substandard or ineffective responses (Apollo & Mbah 2022; Mitchell et al. 2008). Women’s groups also perform a vital role in ensuring community survival and recovery (Alburo-Cañete 2024; Gordon 2013; Pineda et al. 2016; Yoshihama & Yunomae 2018). Thus, instead of restricting humanitarian conversations to authorities and humanitarians, opening up these conversations to others beyond the formal humanitarian space can yield valuable insights and contribute to more effective crisis response.

Humanitarian observatories have been designed based on these three insights. They are networks of diverse stakeholders (including but not restricted to the triangle of authorities, humanitarians, and affected communities) that seek to exchange knowledge, learn, and advocate to reform humanitarian policy and practice in their context. In what follows, we highlight the processes, insights, and lessons from establishing humanitarian observatories in different crisis settings.

## Establishing a network, building a movement

Humanitarian observatories originate from the Hum-Gov research project that aims to understand the evolving landscape of humanitarian action, with a particular focus on perspectives of affected people and civil society actors in crisis settings. Investigating the dynamics between international actors, national authorities, civil society, and affected populations, the project identifies existing and/or alternative humanitarian practices across three case studies in Ethiopia, the Democratic Republic of Congo (DRC), and Colombia. According to the project lead, Dorothea Hilhorst, as follows:

When we were working on the Hum-Gov proposal we wanted to establish networks in the countries of research. This was initially conceptualized to support the ethical commitments of the project. As academics we are always ready to criticize the humanitarian system for not being respectful to national actors, and we wanted to walk the talk in our own research practice.

I discussed the idea of the humanitarian observatories with the research partners in each country and, encouraged by their responses, entered them in the research proposal. I could not have dreamed how the initiative was going to come to life. Although the initiative is still young, the observatories are turning into platforms that shape knowledge and promote reforms in humanitarian governance.

As indicated, the idea of setting up humanitarian observatories was intended to support research activities of the project: as a means to explore context-specific approaches to undertaking research in country case studies, validate frameworks employed by the project and its results, and solicit informed views on the strengths, weaknesses, and alternatives for humanitarian governance in diverse contexts. However, as the Hum-Gov project progressed, the research consortium recognized the potential for the observatories to shape knowledge and promote reforms in humanitarian action as evidenced by advocacy initiatives that were being led by observatory members: in the DRC, the observatory proposed improvements to humanitarian codes of conduct to address sexual exploitation and abuse (Milabyo & Lusambya 2023). In Ethiopia, it advocated for the inclusion of internally displaced people in the peace process (Hordofa 2023) and addressed aid diversion through an advocacy note submitted to the Ethiopian government. The observatory in Colombia focused on the role of civil society and affected communities in humanitarian action and governance in Latin America.^5^

These developments led to discussions around expanding the role of the observatories beyond the original research project towards becoming a movement for inclusive and collaborative knowledge-building and advocacy in the humanitarian field. Due to the initiative’s positive reception among the first observatories, other multisectoral actors from outside the research project areas expressed interest in establishing their own humanitarian observatories. As indicated in the introduction, there are now 11 active observatories. These observatories span different geographical regions and represent a spectrum of complex humanitarian crises including conflict, internal and cross-border displacement, and natural hazards-related disasters at the intersection with development challenges.

Without a fixed formula to how observatories are organized (some are hosted by academic institutions, others by civil society organizations, and others were adopted into already-existing networks), involved actors collectively determine their agendas and find the appropriate ways with which to discuss and reflect on trends and issues relevant to humanitarian practice within their settings. This was done to align with commitments to nurture equitable models of partnerships and minimize the power imbalances discussed above. The Hum-Gov research consortium that enabled the initial establishment of the observatories now takes a more supportive and facilitating role, while the observatories continue to develop, self-govern, address agendas set by their participants, and engage with others within the group.

While rooted in country or regional contexts, observatories also form an international knowledge and advocacy network enabling exchanges and collaborations that cut across geographic areas. Periodic inter-observatory meetings are held to facilitate these exchanges as a means for collective learning, co-reflection, and exploring joint activities and advocacies.^6^ For instance, the observatory in South Asia linked up with the observatory in the Philippines to produce reports and reflections on experiences of heatwaves. They also share experiences of various advocacy initiatives and their strategies during network meetings. In recent months, collaboration has extended to joint initiatives including a proposal for funding drafted jointly by the observatories in DRC and South Asia to research possibilities for achieving heatwave adaptation.

Below, we distil some of the experiences and lessons from the humanitarian observatory initiative. We do this by way of conversation among the authors — all involved in the humanitarian observatories from the beginning — around the experience of working on this initiative. Here, we share reflections on the process of setting up the observatories, their potentials for reforming humanitarian governance, and current challenges of developing this movement. Presenting these points in this manner allows us to keep close to the spirit of engagement and exchange, which has been central to the creation of the network. Moreover, as we are all embedded in the process of movement building through the humanitarian observatories network, it is not possible to detach ourselves from such discussion. Thus, we have instead decided to recognize our own embodied presence in network building, aligning with research approaches that recognize the situatedness of the knowledge we produce and researcher entanglements with one’s subject of study (Haraway 1988; Harding 1993). This conversation is an online exchange conducted in June 2024. Through this discussion, we underscore the insights gleaned for advancing humanitarian knowledge and reform “from below.”

Before proceeding, we first present a brief profile of those involved in the conversation and their roles and activities within the humanitarian observatories network. Dorothea Hilhorst is the lead of the Hum-Gov project. Kaira Zoe Alburo-Cañete is a member of the Hum-Gov research team and has worked closely with the observatories in building the network. She is affiliated with the humanitarian observatory in the Philippines, launched in August 2023. Patrick Milabyo Kyamusugulwa initiated the observatory in DRC that was launched in October 2022. They currently have 25 members, organized several meetings and research missions, and established a website.^7^ Members have written two blog posts and several statements to advocate for support for local disasters. Tadesse Kassa Woldetsadik initiated the observatory in Ethiopia. Officially launched in November 2022, the observatory has conducted multiple workshops on internal displacement law and policy, organized advocacy sessions and published statements on aid diversion and accountability, and written a blog post on inclusion of IDPs in the peace process.^8^ Lastly, Juan Ricardo Aparicio is a founding member of the observatory in Latin America and the Caribbean (LAC), based in Colombia. Recognizing the shared colonial, postcolonial, and decolonial histories of Latin American and Caribbean societies, the observatory took on a regional form rather than remain country-specific. The LAC observatory was thought of as an “experiment” at creating regional, South-South dialogues on issues relevant to humanitarian governance.^9^

## Exchanging experiences and insights: a conversation

**Dorothea Hilhorst (DH):** At the time when we wrote the proposal, we did not know each other. I only knew Patrick very well, as he was my former PhD candidate. I remember you were all polite and enthusiastic. But now I am curious to hear what your thought were at the time. Why were you interested in participating in the initiative? What were your experiences of setting up the observatories like?

**Patrick Milabyo Kyamusugulwa (PMK):** When I heard about the idea of humanitarian observatories, I was attracted by the core idea of promoting humanitarian governance in the Democratic Republic of Congo (DRC), especially in the eastern side of the country. I expected to better understand how humanitarianism works in our country and, through the humanitarian observatory, to contribute perspectives on how to improve it while delivering aid in humanitarian contexts. Other [observatory] participants in DRC are motivated by the same sentiment of better understanding how humanitarian action works in practice and how to improve aid delivery in terms of enhancing social accountability among stakeholders and engage with advocacy on behalf of affected community members towards state and non-state actors.

In DRC, the interplay of conflict and a weak government with natural hazards results in many disasters that do not reach the news but are deadly and devastating in their area. The observatory is an opportunity to know what happens and advocate for assistance. For this, we regularly organize fact-finding missions, for example, after the floods that affected Kalehe with at least 500 deaths in the South-Kivu province alone. Despite the high death toll, the disaster did not attract any attention outside of the area, so we organized the mission to be able to advocate for the victims.

**Tadesse Kassa Woldetsadik (TKW):** For me, the very idea of engaging in the launch and subsequent activities of the Humanitarian Observatory—Ethiopia [HO-Ethiopia] initiative is predicated on the enthusiastic expectation that there must be a robust, fairly independent and well-informed platform in Ethiopia—a country with a plethora of humanitarian actors, programs, and interventions. The observatory is not meant to only observe or analyze trends—but also to debate and concretely inform advocacy for reform of humanitarian governance [in Ethiopia]. I believe the wide range of professionals involved in HO-Ethiopia are enthused by similar considerations; this could be deduced from the observations they raised and suggestions made during the launching ceremony and rounds of observatory forums. Initially, as we were preparing for the launch of the Ethiopia observatory, we conducted interviews with individuals and experts from diverse backgrounds, including officials of the federal government working in relevant offices that respond to crises or coordinate humanitarian responses.

As we progressed with the initiative, due to the controversial nature of humanitarian endeavors in Ethiopia, we discussed if the state officials’ engagement as members might bar some critical debates from happening during the observatory sessions. Humanitarian action in Ethiopia is often criticized to have been very controversial, and we want members to speak freely and confidentially without any fear of real or perceived push-back from the state. For this reason, in a bid to continue providing [a] free and secure space for the participants, we decided against government participation — although we would always subsequently share the outcomes with the government. As discussions on humanitarian space in Ethiopia are often controversial, we also agreed to be governed by the Chatham House Rules, and do not reveal the identity of the member who made any specific comment. Further, to enhance the openness of our discussion, we agreed that members participate in the observatory activities in their individual capacities, and any specific comment they make may not be attributed to the organization to which they are affiliated.

**Juan Ricardo Aparicio (JRA):** Research on humanitarianism as a main theme in Colombia had been almost absent until the [Hum-Gov project]. Indeed, there are tons of large and short reports of NGOs, United Nations, Colombian Government and other international organizations on the problems, situation and conditions of the so-called protracted and contingent humanitarian crisis. There are also very robust research agendas present in books, articles, major academic conferences that have also nurtured graduate and undergraduate academic programs in different universities in Colombia on Peace Reconstruction, Development, Human Rights and other similar dimensions of vulnerability, armed conflict and disaster scenarios. But there was no research explicitly on humanitarian governance. Neither was there a platform for dialogue and exchange between academics, practitioners and organizations that followed the trends and characteristics of the humanitarian dynamics in the country. And much less research that also wanted to intervene in the public discussion with relevant actors on the design of humanitarian actions. There were only a few of us in Colombia that ethnographically examined humanitarian regimes, their discourses and practices, their relations with power structures, states of exceptions and historical dimensions, both from the headquarters offices but also in the daily interactions of unfolding humanitarian actions. And then the project and the idea of the Humanitarian Observatories arrived to radically change this history.

**Kaira Zoe Alburo-Cañete (KAC):** I really like how all of you emphasize, in different but convergent ways, the importance of understanding humanitarian action in context. This made me think of how the approaches for establishing the observatories are also contextual. For example, when I was facilitating the setup of the observatory in the Philippines, the group leading the initiative, Center for Disaster Preparedness, did not feel the need to put in place a separate entity for the observatory. For other observatories, there was a need to organize the network of actors and even formalize as a group such as the case of DRC. But in the Philippines, there was already an existing network and modality for dialogues that could incorporate the functions of a humanitarian observatory. In this way, the observatory became an add-on feature to what was already operating as a multisectoral network of national actors involved in, but not exclusively, humanitarian practice. So, I find it really interesting how even the idea of humanitarian observatories can be applied in different ways, in different contexts, and according to what organizations comprising each observatory deem appropriate. On this note, perhaps another question we could all reflect on is the nature of the partnership we are creating through the humanitarian observatories. There have been many critiques about how humanitarian knowledge-building practices tend to replicate inequalities in the current global system (see Hilhorst 2020). What are your thoughts on these and how do you think the humanitarian observatories help address these issues?

**JRA:** For me, taking part in the observatories is not only about making visible a ‘new’ research agenda. It is also about creating and promoting a different way of producing knowledge between academics working in the Global North and South and I am aware of all the problems that these categories mays have. Normally, this relation historically has meant that ‘we’ in the South produce ‘facts’ while ‘theory’ and ‘research design’ is produced in the North. Colleagues of the South are recruited to find ‘facts’. But the [Hum-Gov] project and the Observatories radically breaks with this tradition of a very pervasive geopolitics of knowledge that is also racial, gendered, epistemological and ontological and that has also been researched by a tradition of critical Latin American and Caribbean thought. The project and the Humanitarian Observatories were designed in a very complex and interesting way where there is still centralized coordination but where the interaction with colleagues of the selected countries is radically horizontal. In this complex maze of dialogues and decision-making processes, for instance, we in Colombia decided that the Observatory should be regional and not country-focused. We decided and we didn´t have to ask for permission or authorization. It was our call, and we had our reasons to do it. Also, with the rationalities and justifications for recruiting academics, practitioners and other people coming from the NGOs and other organizations. Also, with the themes and organizational dynamics of our Latin American and Caribbean Humanitarian Observatory. I have always been surprised with the repeating answer to our decisions: “it is your call, you decide [if]you can and should do it”.

**PMK**: In the case of the DRC, I can account how we were able to write our own blog on “Adapting codes of conduct for humanitarian workers to the DRC context can prevent and combat sexual abuse” The idea followed the group discussion we had in our Humanitarian Observatory (HO) on how widespread sexual abuse is ravaging the Democratic Republic of the Congo’s humanitarian sector. We realized that context matters even though there exist the old humanitarian code of conduct, its application varies from one zone to another zone, not to mention how the perception of the code intersects with local norms and interest people have to benefit from aid or join international organizations or United Nations Agencies at least as temporary staff for the sake of survival. The idea was self-driven by the DRC HO, we decided to discuss the issue on 15 May 2023 in three group discussions and I remember that members brought in the discussion each its own idea that led to the writing of the blog. Ideas to translate the code in local languages and local dialects, to raise awareness on it among community members and to involve local actors including state officials in its application. There was no influence from The Hague to produce the knowledge, we were ourselves actors to produce facts and knowledge. So, it really depends on whether a space is created as I see it within observatories for co-creation of both facts and knowledge regardless where one is situated whether in the global North or in the global South.

**DH:** The enthusiasm for the observatories speaks from your answers and was obvious from the start. I am also truly impressed by the many activities and outputs that have been achieved. Even though your observatories are less than 2 years young, are you confident that your expectations will be met?

**TKW:** Since the launch of the national chapter in November 2022, we were able to hold four rounds of observatory dialogues on various themes; and collaborate with other observatories on issues of common concern such as conflict and refugee crises. Starting with a modest number (15), our membership has continuously grown over the years and we are receiving applications from individuals working in local and national organizations to join the observatory. For me and other members, the four events and follow-up actions have proved critical platforms—providing a long-sought ‘independent’ space for discussing, debating and exchange of views on vital, and in most cases controversial, topics of humanitarianism in Ethiopia. To such extent, the observatory platform has met my expectations. I am confident that the observatory will continue to provide alternative views on the way how responses to humanitarian crises in Ethiopia should be organized or reformed.

**PMK:** Our expectation has started to be met as we have started to collect regular data on humanitarian activities in eastern DRC; then, we have started to engage with stakeholders in advocacy notes and activities such as advocacy café in relation to humanitarian crises after massive flooding in Kalehe and Bukavu in the South-Kivu province as well as massive internal displacement around Goma in the North-Kivu province. Members are united and are very much motivated by the idea of bringing our own contribution to trying to solve governance issues in our country, in particular in relation to social accountability, aid effectiveness and advocacy. Since October 2022, members have published 2 online articles related to sexual abuse in the humanitarian sector and have approved statutes and internal rules of our DRC Humanitarian Observatory in the perspective to sustain it and its activities.

**JRA:** Perhaps one of the most obvious successes of the Observatory has been to bring together scholars, practitioners and affected communities that would seldom know about each other and that could continue to work in isolation. With reading groups, a website, newsletters, blog, frequent meetings and an edited volume in process, the Observatory has turned into an authentic place of exchange of ideas taking place on a common ground and with shared vocabulary. It has turned into a community of knowledge, care and support for diagnosing humanitarian issues in the region while imagining other alternatives away from the top-down initiatives.

**KAC:** It’s great to hear about these activities progressing. I am also quite interested in knowing more about the challenges you are encountering in running the observatories, or some issues that you foresee might need deeper consideration. Would you share your thoughts on these? And more importantly, can you give some examples of how you have navigated these challenges and what strategies you have put in place to address them?

**PMK:** Efforts for change are always associated with challenges. When we started, there were more people, we noticed the diminution of participants (from 25 to 15) in events. With progress made in terms of regular meetings, adopting the HO statutes, collecting evidence before engaging in advocacy actions, interesting themes of discussions such as floods crisis of Kalehe, massive displacement around Goma, some of members who were hesitant have returned. Also, there are new members who are interested in our activities. Another challenge is difficult collaboration with UNOCHA in Bukavu as at one time we were allowed to share a presentation of one our findings in a UNOCHA meeting, later we noticed hesitance on that decision. We are planning to have a joint presentation with one international organization well known here (ZOA) to be able to do so. Last, in our advocacy café,^10^ some key stakeholders did not show up, namely representatives of United Nations agencies based in Bukavu and the Mayor of the city. We were able to share the report about the activity with them in their respective offices after the event.

**JRA:** A major concern that we are having at this moment is on the dependence of the LAC Observatory on the presence of a few members; and in general, on the Hum-Gov project. In different ways we have told other members that the Observatory is a common platform and that we truly will welcome a more active participation on its decision-making process and rationalities. We are afraid that this dependence can hinder the sustainability and regional scope of this important initiative. Also, although we do believe that academy can play a key role in the rigorous research agendas needed to follow the complexities of the humanitarian arena, we are also aware that the academy moves in a different rhythm than afflicted communities and humanitarian organizations. We do research, write, and publish journal articles and books. This is a process that takes time and also long-term engagement with communities that also have their own agencies and priorities while organizations heavily rely on a recipe knowledge. This location in the academy is one in which we must reflect constantly. We are relatively slow and we want to avoid reproducing extractivist knowledge practices.

**TKW:** I think that there are a few outstanding matters that merit thoughtful consideration. First, membership expectation is fairly high in terms of generating impact; as much as advocacy work on diverse issues of humanitarian action constitutes a key pillar of our undertaking, we must also be pragmatic in terms of how we coin approaches and languages. It is important to note that Observatory members are drawn from professionals, academics, researchers, and representatives of civil society that bring in diverse (and at times aggressive or incompatible) perspectives on issues pertaining to people at risk. Secondly, I think it is one thing we possess valuable observations for at-risk populations—and our observatory will certainly play role in soliciting informed views on the strengths, weaknesses and alternatives of humanitarian governance in Ethiopia; but, a question remains how (or what approach we need to deploy) to meaningfully influence discourse and practice in Ethiopia that is significantly dominated by donor agencies and government machinery (in a political economy that hosts very fewer proactive civil society actors). All in all, I believe we have come a long way from mere theoretical insights—and yet, as we go along, we would also need to have further clarity on how we delineate and promote the modalities of operation of the Observatory so that expectations are fully met.

## Elucidating elements of movement building

From this conversation, it is clear that the process of building the humanitarian observatories network is not straightforward. As with many social movements — and indeed, we see the network as comprising a movement for pursuing transformations in the humanitarian field — the workings of the humanitarian observatories are a mixture of organization and spontaneity. They align under a common purpose: to generate and advance knowledge from contexts affected by crisis and to identify points for advocacy and action and move them along. However, they differ in terms of their strategies, priorities, focus, and formations. They move independently but to some extent are also coordinated. Our conversation elucidates aspirations for reform, pursuits for meaningful partnerships while navigating the potentials of (and the tensions that come with) multi-actor engagement — all of which take shape in practices of “learning by doing” as well as “doing by learning.”

One of the most prominent qualities of the humanitarian observatories is their self-driven and self-governing nature. In the preceding dialogue, this point comes through clearly in how we have spoken about being able to determine each observatory’s agenda with autonomy. This is a stark contrast from previous experiences of partnerships wherein “donor” and specifically global north-based organizations get to decide activities and goals. Above, J. R. A. expresses surprise at the sense of autonomy they experienced in running their observatory precisely because this model of partnership is not very common.

The ability to work autonomously enables the valuing of embodied knowledges and experiences. It helps address the problem of knowledge inequities identified in the previous sections of this article that come about because of power imbalances. P. M. K. succinctly captures this when he said “[t]here was no influence from The Hague to produce the knowledge, we were ourselves actors to produce facts and knowledge.” More importantly, it becomes a platform to develop alternative views on humanitarian practices and governance, as T. K. W. pointed out.

By being able to explore, reflect, and analyze issues present in their own context, humanitarian observatories also enable the creation of context-specific strategies for addressing issues and challenges. For example, T. K. W. talks about how the atmosphere of fear and mistrust around the topic of humanitarian response affects the ability to facilitate meaningful dialogue. The observatory in Ethiopia therefore had to devise strategies to enable discussion between multiple actors, especially when engaging with government. K. A. C, meanwhile discusses how the Philippine observatory leverages its strong connection with civil society networks in the country to pursue its objectives. Lastly, the DRC observatory has adopted an evidence-based approach to humanitarian advocacy: by conducting data collection and fact-finding missions and organizing advocacy cafes to enable stakeholder engagement.

This attunement to context and active development of appropriate strategies to navigate these diverse settings enable observatories to become hubs of innovation and creativity. This is evidenced by the variety of outputs they have produced thus far. Drafting of advocacy notes, holding multistakeholder meetings, production of academic books and articles, publishing blog posts for public dissemination, and organizing webinars on pertinent issues are just a few examples of outputs of the individual observatories within the last couple of years. While we have underscored here the independent initiatives of the humanitarian observatories, it is important to recognize another quality of the humanitarian observatories that adds to its value as a platform for knowledge generation and advocacy: inter-observatory engagement, learning, and exchange. At least twice a year, humanitarian observatories come together to share experiences, challenges, and insights, as well as to explore collaborative projects. For instance, during the November 2024 inter-observatory meeting, the network organized a working group to develop joint initiatives, among other action points identified. In this way, the inter-observatory network meetings become a site for “cross-pollination” — enabling collective thinking around how best to achieve impact in the humanitarian field.

Living up to the values of the observatories as autonomous spaces was not without challenges. The Hum-Gov project needed to reconcile the values of the observatories with the requirements of project administration that, for example, conditions funding on previously agreed deliverables. This particular requirement was accommodated by providing the observatories a free hand in listing the deliverables that were incorporated in the agreements. Similar hurdles were taken by revisiting the values in conversation with the observatory coordinators.

Although there continues to be much enthusiasm around what the humanitarian observatories can attain, its members are also aware of the challenges of sustaining such a movement. One point of caution, as raised by JRA, pertains to dependency on a small core of leading actors that seems an inevitable aspect of new initiatives. PMK and TKW also underscore the need to plan and strategize how to meaningfully influence humanitarian discourse, practices, and governance. Indeed, the humanitarian observatories have been able to produce different outputs and to a certain extent have engaged in advocacy work with multiple actors. However, as tabled in the last two inter-observatory meetings in March and June 2024, deeper discussion needs to ensue to make sure that there is durable uptake of the knowledge produced from their activities. Finally, sustainability of any movement also depends on availability of resources and funding. All observatories utilized seed money. The frugal use of this seed money has been one of the tokens of buy-in, as there are no salaries involved and all activities have been voluntary or sponsored by the host organizations. The seed money is thus stretched as long as possible but will nonetheless be depleted at some time. While there are no clear-cut solutions to these issues, the observatories network is actively finding ways to address these challenges, for example, through the development of working groups, collaboratively writing project proposals, as well as undertaking individual initiatives for fundraising and acquiring institutional support. A recently obtained funding for the observatories will be used to roll out completely flexible “micro grants” for individuals or groups associated with observatories.

**Fig. 1:**
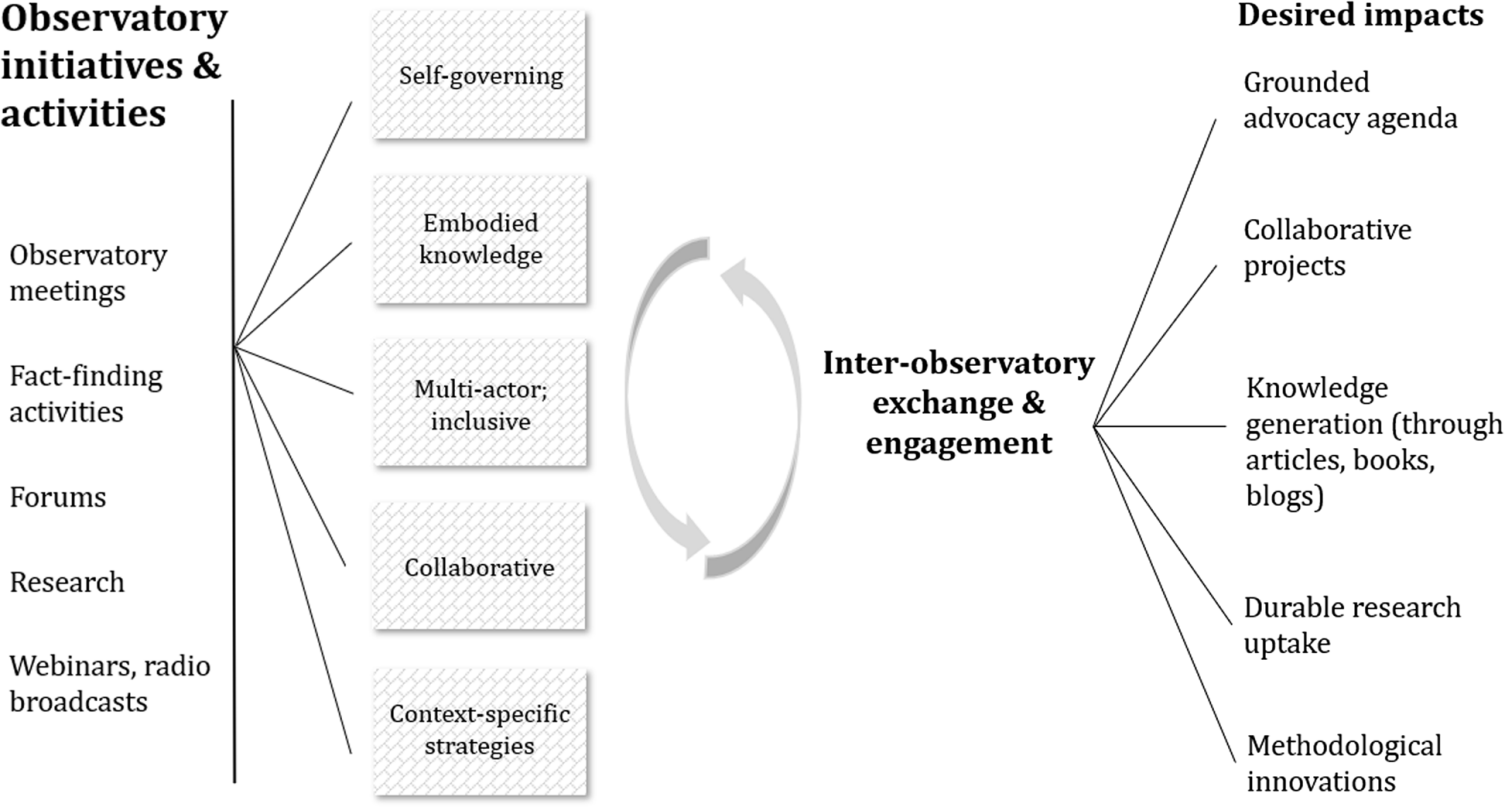
Humanitarian observatories’ qualities, guiding principles, and processes

Overall, the humanitarian observatories can be considered a work in process. Their development is non-linear, uneven, and iterative. They have shown innovation, creativity, and reflexivity while navigating challenges that come with movement building. The diagram below summarizes some of the insights and lessons that have emerged from this exercise of conversation and co-reflection (Fig. 1).

## Concluding remarks

This paper sets out to discuss the nascent “movement” of the humanitarian observatories. The first observatories were launched in 2022, and they are still unfolding and finding their shape, ways of working, and possible impact.

There are currently many initiatives that focus on organizing and amplifying the views and capacities of actors in the Global South in relation to humanitarian action. Such initiatives include, for instance, the NEAR network and the START Network’s creation of humanitarian “hubs,” which are collectives of multiple humanitarian actors present in a crisis-affected country established to enable better decision-making and coordination.^11^ Rather than reviewing these different initiatives and their specificities, this article has focused on the humanitarian observatories and their potential contributions to achieve reforms. There are a few aspects of the observatories that stand out and may be inspiring to or can be considered in these other ongoing initiatives.

First, the notion underpinning the observatories that humanitarians are not the only relevant actors to deal with humanitarian crises goes out to be a major strength. In the Philippines, the humanitarian observatory is an add-on of an ongoing network. Asked about the added value of being an observatory, the coordinator immediately stated the following: “that the observatory has a diversity of members.” The academics, NGOs, community-based organizations, and journalists that are part of the observatories next to humanitarians working for national or international agencies are equally motivated to consider issues of humanitarian action and bring a diversity of views and methods to the observatories.

Second, context matters indeed. Not only do the observatories deal with different topics — sexual violence and abuse in DRC, aid diversion and internal displacement in Ethiopia, and the role of communities in Latin America, but also they markedly differ in their approaches and styles. The Latin American Observatory has a tendency to focus on the intellectual contributions of the continent to humanitarian studies, with a monthly reading group and a number of publications in the pipeline. In Ethiopia, a delicate balance is being sought between critically discussing the politics of aid and providing feedback to the government in ways that do not jeopardize relationships. In DRC, the observatory has developed a hands-on modality of fact-finding missions followed by focused advocacy. The members of the observatory have a messaging app group in which they share news of disaster events, like dreadful fires in urban poor areas. Their advocacy café involved neighborhood chiefs to talk about measures they could take in case of heavy rains.

Third, it is striking how highly appreciated it is that the observatories set their own agendas. All partners are used to initiatives that are grounded in a language of partnership yet where the sponsoring party dictates deliverables. This requires continuous negotiation about the administrative practices of a university that are geared to implanting set goals and achieving timely deliverables. In the case of the observatories, contracts mention deliverables because that is a condition from the university that manages the fund. However, the addendum on deliverables is entirely drafted by the observatories, and they are urged not to overpromise on their outputs — ensuring that the “deliverables” they identify truly reflect their interests, capacities, and aspirations. Even though the frustrations of “Southern Partners” in this respect are widely known (Haar & Hilhorst 2009; Villacis et al. 2022), it is nonetheless striking how strong the relief is when this is not the case and how this self-agenda setting engenders creativity and contextually rooted insights and activities.

Fourth, one of the most interesting aspects of the observatories is the sideway interactions. Members of observatories have been invited to join activities of other observatories, and Ethiopian participants have, for example, taken part in a discussion about the situation in Sudan organized by the South Asian observatory. Twice a year, observatory members meet to exchange experiences and compare notes. In the last meeting of June 2024, one of the main action points identified was to organize a focused learning session on designing advocacy strategies, drawing on the expertise of members within the network. As one of the members stated in a recent inter-observatory meeting: “I like the idea of sharing experience among different observatories. For example, there are observatories with experience in advocacy that other can learn from. We are diverse [with] different cultures, so it is not about “copy-paste.” Each observatory should be able to learn from others and try to do what is appropriate in their context.

It is yet to be seen in what ways the observatories will indeed contribute to reform from below. Observatories are differential in their access to the usual players of the international humanitarian community, but in general, there are no signals yet of productive interactions with these players. In DRC, as elaborated above, the observatory has made many efforts to present its work in one of the cluster meetings which still needs to materialize. In other observatories, employees of international agencies prefer to participate on a personal title as they feel they can otherwise not speak out freely, and they are not in a comfortable position to report findings of the observatory to their organization. However, the question of reform cannot be reduced to whether or not international humanitarian players listen and act on findings. It relates to complex questions on how reform is triggered and how futures of humanitarian action can be imagined. How this can happen is beyond the scope of this paper and too early to discuss for the case of the observatories.

## Data Availability

Not applicable.

## Footnotes

1 https://www.ifrc.org/our-promise/do-good/code-conduct-movement-ngos

2 See https://www.shiftthepowersummit.org/weavingconversations.

3 https://ihsa.info/expertise/map/

4 *Humanitarian governance: accountability, advocacy, and alternatives*, financed by a European Research Council (ERC) advanced grant.

5 See https://cienciassociales.uniandes.edu.co/observatorio-humanitario-de-america-latina/.

6 The first inter-observatory meeting was in March 2023, hybrid format and held in Colombia, hosted by the regional observatory there. The second meeting was done face t -face in Bangladesh, during the 2023 International Humanitarian Studies Conference in November. The next two meetings (June and November 2024) were done online.

7 https://ohrdc.org/

8 BlISS, the official blog of the International Institute of Social Studies, runs a blog series that features the work of the humanitarian observatories. This serves as a platform for communicating research and perspective pieces developed by observatories. The blog series can be found here: https://issblog.nl/humanitarian-observatories-series/

9 The Humanitarian Observatories Network publishes newsletters that outline its major activities. The latest newsletter can be accessed here: https://www.humanitarianstudiescentre.nl/humanitarian-observatories/

10 The advocacy café is one of the innovations developed by the DRC HO, serving as a platform to provide spaces for multistakeholder/sectoral discussions with the intent of developing and pursuing specific advocacy agenda for humanitarian action.

11 https://startnetwork.org/network/hubs
